# Atypical Bilateral and Unilateral Vocal Cord Paralysis in Two Neonates With Williams Syndrome

**DOI:** 10.1155/crpe/8647206

**Published:** 2026-05-27

**Authors:** Priya Arya, Miller C. Hamrick, Benjamin Mackowiak, Bradley Buckler, Chad Haldeman-Englert, Stephanie E. Ambrose

**Affiliations:** ^1^ Mercer University School of Medicine, 1250 E 66th St, Savannah, 31404, Georgia, USA, mercer.edu; ^2^ Department of Pediatric Surgery, Children’s Hospital of Savannah, 4700 Waters Ave, Savannah, 31404, Georgia, USA; ^3^ Department of Neonatology, Children’s Hospital of Savannah, 4700 Waters Ave, Savannah, 31404, Georgia, USA; ^4^ Department of Medical Genetics & Genomics, Fullerton Genetics Center, 9 Vanderbilt Park Dr, Asheville, 28803, North Carolina, USA, unc.edu; ^5^ Department of Pediatric Otolaryngology, Children’s Hospital of Savannah, 4700 Waters Ave, Savannah, 31404, Georgia, USA

**Keywords:** bilateral vocal fold paralysis, oropharyngeal dysphagia, unilateral vocal fold paralysis, Williams syndrome

## Abstract

Williams syndrome is a rare genetic disorder occurring in approximately 1 in 7500 individuals. The condition occurs as a result of deletions within chromosome 7q11.23, manifesting a unique phenotype with a wide constellation of symptoms. In this report of two cases, we present the care of two early‐term infants with Williams syndrome. The first infant was noted to have bilateral paralysis of the vocal folds and required multidisciplinary care that ultimately mandated home tracheostomy and gastrostomy tube usage, while the second had unilateral vocal cord paralysis and similarly needed continued gastrostomy tube usage and feeding therapy. This dysfunction of the vocal folds was thought to have occurred due to the deletion of the gene region involving elastin, resulting in these atypical findings that increased the complexity of care of these patients. We document the clinical courses of these patients and their treatment regimens.

## 1. Introduction

Williams syndrome is an uncommon disease, first described in 1961 with an incidence of 1 in 7500 reported in the literature [1]. A heterozygous chromosome 7q11.23 deletion in the Williams syndrome critical region (WSCR), with a length of 1.55–1.8 Mb, engenders a phenotype characterized by craniofacial abnormalities, intellectual disability, connective tissue differences, infantile hypercalcemia, and a highly social, friendly, and distractable behavioral profile [2]. The syndrome not only usually occurs sporadically but can also be inherited in an autosomal dominant fashion [3]. Williams syndrome patients require complex, multifaceted care, involving numerous medical and surgical disciplines including but not limited to pediatric surgery, otolaryngology, neurology, cardiology, endocrinology, and gastroenterology.

In this case report, we detail the course of hospitalization for two patients with Williams syndrome and vocal fold palsy, postulated to be due to the involvement of the gene region encoding elastin and adding a further level of complexity to their care.

## 2. Case Report 1: Bilateral Vocal Fold Paralysis

Patient 1 was a female born vaginally at 38 weeks to a 34‐year‐old G3P2 mother. The pregnancy was complicated by polyhydramnios with possible IUGR. There was no history of abnormal maternal screening or teratogenic exposures. Upon delivery, the infant sustained a right clavicular fracture and presented with stridor, respiratory distress, and suprasternal and subcostal retractions.

**FIGURE 1:**
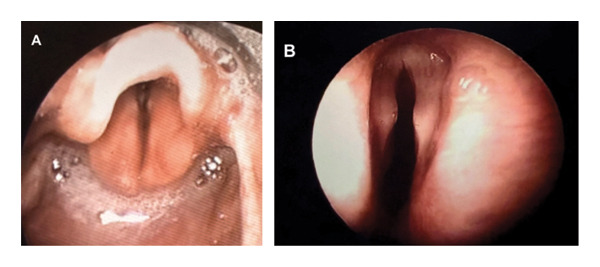
(A) Flexible fiberoptic laryngoscopy demonstrating vocal fold paralysis. (B) Bilateral vocal fold immobility was seen in Case 1, with minimal abduction during inspiration.

The infant was admitted to the neonatal intensive care unit with suspicions of laryngomalacia, tracheobronchomalacia, or a fixed obstruction such as a vascular web or ring. Otolaryngology was consulted, and flexible endoscopy revealed reduced abduction of vocal folds bilaterally, consistent with bilateral vocal fold paralysis (Figure 1A,B). The patient was initially monitored and started on airway dexamethasone due to suspected paralysis secondary to birth trauma, but the patient ultimately required intubation. Further workup in the form of operative microlaryngoscopy and bronchoscopy revealed no acute findings and mobile cricoarytenoid joints. MRI revealed multifocal areas of hemorrhagic white matter injury bilaterally in the cerebral hemispheres but showed no evidence of Chiari malformation. Echocardiography revealed patent ductus arteriosus, supravalvular pulmonary stenosis, bilateral peripheral pulmonary stenosis, and patent foramen ovale. Magnetic resonance angiography recommended by neurology to rule out a vessel dissection was unremarkable.

The patient was extubated to noninvasive positive pressure ventilation following steroids, but subsequent bedside bronchoscopy revealed continued vocal fold immobility. Subsequent increased work of breathing and retractions were noted, and the patient ultimately required tracheostomy, Nissen fundoplication, and gastrostomy feeding tube placement. The postoperative course of the patient was complicated by need for high‐frequency oscillatory ventilation (HFOV), eventually weaning to assist‐control ventilation, tracheal CPAP, and ultimately room air.

Given the unexplained vocal fold paralysis and cardiac abnormalities of the infant, a chromosomal microarray was sent. Results showed a heterozygous chromosome 7q11.23 deletion (chr7:72,745,047‐74,138,459, hg19) in the WSCR, consistent with Williams syndrome. Additionally, a terminal chromosome 17p13.3 duplication was revealed (chr17:48,858‐624,525, hg19), considered an uncertain variant. Parents of the patient were counseled that vocal cord paralysis is not a commonly seen feature of Williams syndrome but has been previously described and likely related to abnormalities of the elastin gene, found within the deleted region. Video swallow under fluoroscopy and renal ultrasounds both had unremarkable findings. Ophthalmology consult was unremarkable, noting mature retinas but acknowledging it to be too early to rule out strabismus. Endocrinology was consulted per thyroid lab abnormalities noted in patient, with elevated TSH of 6.60 uIU/mL. Free T_4_ levels were normal at 1.74 ng/dL. After one week of an uncomplicated hospital course, the patient was discharged home with tracheostomy and heat moisture exchanger (HME), as well as feeding tube in place.

The patient was admitted two weeks later for increased work of breathing. Bacterial tracheitis was suspected, and cultures confirmed bacterial infection secondary to *Staphylococcus aureus* and *Stenotrophomonas*, as well as viral upper respiratory infection (URI) secondary to rhinovirus. The patient was administered ciprofloxacin drops, and she proceeded to recover. The patient is now 5 months of age and continues to follow with pediatric gastroenterology for her oropharyngeal dysphagia, feeding recommendations, and monitoring for appropriate weight gain, amongst other pediatric specialists.

## 3. Case Report 2: Unilateral Vocal Fold Paralysis

Patient 2 was a male born at 37 weeks by repeat cesarean section to a 29‐year‐old G2P2 female. Complications of the pregnancy and delivery included pre‐eclampsia and macrosomia, requiring three vacuum extraction attempts. The infant experienced respiratory distress after delivery requiring CPAP, surfactant, and eventual intubation to facilitate safe transport for higher level of care. He was noted to have a small pneumothorax that spontaneously resolved. He was extubated and weaned to room air by a week of life without further concerns regarding his respiratory status.

**FIGURE 2:**
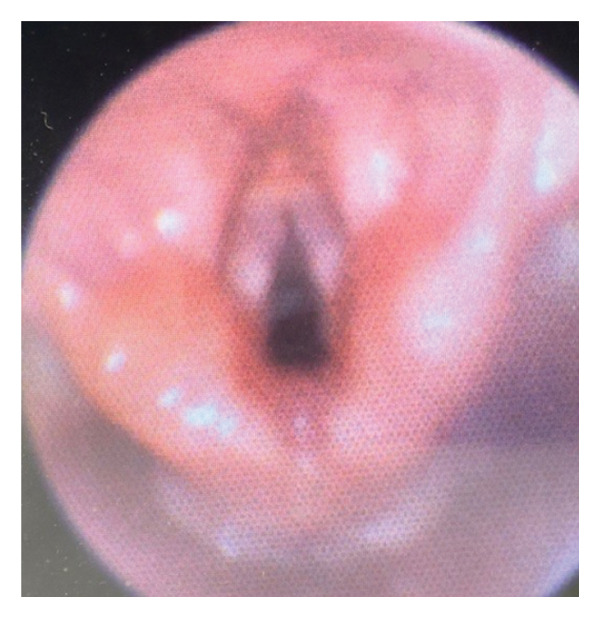
Right vocal fold hypomobility in Case 2, with compensatory left vocal fold movement seen during phonation.

By two weeks of life, the otolaryngology service was consulted due to poor feeding skills, ankyloglossia, and a modified barium swallow study (MBSS) revealing mild nasopharyngeal regurgitation without penetration or aspiration. He underwent lingual frenotomy and was noted to have some mild dysmorphic facial features including mild retromicrognathia, bilateral preauricular pits, and a thin upper lip. He was recommended to continue working with feeding therapy, but by three weeks of life, he continued to experience frequent coughing, despite strict external pacing on all trialed bottles except ultrapreemie nipple. Otolaryngology was consulted again, and bedside physical exam included normal‐appearing palate without evidence of submucous cleft palate and well‐healing frenotomy site. Flexible laryngoscopy revealed a hypomobile right vocal fold (Figure 2). Recommendations were made for repeat MBSS, as well as genetics and neurology evaluations.

Repeat MBSS revealed dysfunctional swallowing with significant velopharyngeal insufficiency, diffuse pharyngeal residue after swallow, and silent aspiration on the posterior tracheal wall. Other diagnostic tests included an echocardiogram, which showed a small patent ductus arteriosus with left‐to‐right shunt, mild‐to‐moderate bilateral pulmonary artery branch stenosis, and mild supravalvar pulmonary stenosis. Repeat echocardiogram revealed bilateral peripheral pulmonary stenosis, patent foramen ovale, and no mention of valvar pulmonary stenosis. MRI of the brain was normal. Initial newborn metabolic screen at the outside hospital revealed normal TSH and low T4 levels at 5.8 ng/dL. Repeat screening revealed a TSH of 6.49 uIU/mL and free T4 of 1.45 ng/dL. Video genetics consultation was performed with recommendation for exome sequencing, which identified a heterozygous chromosome 7q11.23 deletion (chr7:72,717,397‐74,148,352, hg19), consistent with a diagnosis of Williams Syndrome.

The patient then underwent gastrostomy feeding tube placement and Nissen fundoplication at four weeks of life, in addition to continued use of ultrapreemie nipple with feeding therapy. He was discharged and seen at four months of age with repeat flexible laryngoscopy showing full velopharyngeal closure, as well as a persistent hypomobile right vocal fold with adequate compensation from the left vocal fold. He was continuing feeding therapy with oral and gastrostomy tube feeds with plan for interval follow‐up to assess vocal fold function. A direct comparison of the two cases is summarized in Table 1.

**TABLE 1:** Case comparisons.

Feature	Case 1	Case 2
Sex	Female	Male
Gestational age	38 weeks	37 weeks
Delivery	Vaginal	Repeat C‐section (vacuum attempts)
Initial presentation	Stridor, respiratory distress	Feeding difficulty, coughing
Vocal fold findings	Bilateral paralysis	Right unilateral hypomobility
Airway intervention	Tracheostomy required	No tracheostomy
Feeding issues	Dysphagia requiring G‐tube	Aspiration requiring G‐tube
Cardiac findings	PDA, supravalvular & peripheral pulmonary stenosis	PDA, bilateral pulmonary stenosis
Neurologic imaging	White matter injury	Normal MRI
Genetic findings	7q11.23 deletion + 17p13.3 duplication	7q11.23 deletion
Hospital course	Prolonged, ventilatory support	Shorter respiratory course
Long‐term needs	Trach + G‐tube	G‐tube + feeding therapy
Outcome	Persistent bilateral paralysis	Compensation from contralateral fold

## 4. Discussion

Previous reports have been described in the literature of vocal phonation dysfunction in Williams syndrome patients, attributed to the disruption of elastin‐rich tissues in vocal fold structures such as the lamina propria and Reinke’s space [4–6]. Histologic studies of haploinsufficiency of the *ELN* gene in carrier mice, as well as postmortem larynx analysis in a Williams syndrome patient, have revealed a paucity of elastin deposition within the vocal folds [7, 8]. However, it is unclear whether this mechanism only alters the nature of one’s voice, as opposed to causing paresis of the cords entirely.

Although both patients shared deletions within the WSCR, subtle differences in deletion boundaries may partially explain the phenotypic variability observed between bilateral and unilateral vocal fold paralysis. Elastin deficiency may be one of multiple mechanisms to fully explain vocal fold immobility. Genes within the WSCR such as GTF2I and BAZ1B have been implicated in neural development, cytoskeletal organization, and cranial nerve patterning [9, 10]. Variability in the extent of deletion involving these genes may influence vagal nerve development, neuromuscular junction integrity, or central coordination of laryngeal motor function. This could plausibly contribute to asymmetric or complete vocal fold paresis, as observed in these two cases.

The bilateral paralysis in Case 1 may reflect more diffuse neurologic vulnerability or impaired compensatory mechanisms. In contrast, the unilateral paralysis observed in Case 2, with subsequent contralateral compensation, suggests partial preservation of neuromuscular function and central coordination. These observations raise the possibility that vocal fold paralysis in Williams syndrome represents a spectrum of neurolaryngeal involvement rather than a uniform structural abnormality.

For the first infant, this is the sixth case report in the literature of bilateral vocal fold paralysis in a Williams syndrome patient. For the second infant, we were unable to find pre‐existing reports of unilateral vocal fold paralysis with Williams syndrome and are uncertain how many cases exist. Prior reported cases of bilateral vocal fold paralysis include a case described by Stewart et al. of a 9‐year‐old girl with acute bilateral cord paralysis, presenting with inspiratory stridor associated with tracheal tug and subcostal and intercostal recession. The patient was described to have a gruff voice since the age of 3 and ultimately required indefinite tracheostomy placement. The authors presented a possibility that Williams syndrome–associated abnormalities in calcium homeostasis and associated calcitonin/CGRP axis metabolism may play a role in neuronal dysfunction, leading to vocal cord paresis [11]. Takamatsu additionally reported a series of pediatric patients presenting with bilateral vocal cord paralysis, including one with Williams syndrome [12]. Further details of care were not available. Vaux et al. reported two neonatal cases, one of which received a tracheostomy on the fourth day of life and subsequent need for Nissen fundoplication and gastrostomy tube placement, similar to the course of the patient detailed in our report. Their other case had similar bilateral cord paresis in the context of Williams syndrome but was lost to follow‐up [8].

Koren et al. also report a case of a neonate with Williams syndrome presenting with inspiratory stridor, later found to have bilateral cord paresis [13]. He underwent tracheostomy placement and later began to vocalize with a gruff voice, ultimately undergoing a posterior right vocal cordectomy at the age of five in anticipation of future decannulation. Notably, Koren et al. also reported hypothyroidism in their patient, with a TSH level progressing from 1.91 to 7.5 and to 50.5 mU/L by three weeks of age, eventually being started on levothyroxine. Our patient had similar findings, albeit subclinical hypothyroidism, with an elevated TSH and normal FT_4_.

Regarding the additional uncertain 17p13.3 duplication variant of the first patient, a study by Curry et al. describes patients with larger duplications of this region and associated phenotypes as varying but include impaired cognitive development, autism, distinct facies, and structural brain abnormalities [14]. Connective tissue phenotypes are also present in these patients but are more similar to Marfan syndrome. Therefore, we do not suspect that this duplication is related to the phenotype of the patient at this time. More information will help determine the future significance of this duplication.

For the infant in our first case, her bilateral vocal fold palsy added an increased level of complexity to her hospital and home course of care, as she required indefinite tracheostomy placement to secure her respiratory function, as well as usage of a gastrostomy feeding tube. This complication may also have predisposed her to infection, as evidenced by her subsequent hospitalization for suspected bacterial tracheitis and viral URI. In contrast, Case 2 demonstrated unilateral vocal fold paralysis with preserved compensatory function from the contralateral fold. As a result, airway protection was maintained without the need for tracheostomy. However, impaired glottic closure and velopharyngeal dysfunction resulted in significant feeding difficulties and silent aspiration, ultimately requiring gastrostomy tube placement and prolonged feeding therapy.

Across both cases, several core clinical lessons emerge. First, early airway evaluation is critical in infants with Williams syndrome presenting with stridor or feeding difficulties. These patients should undergo prompt laryngoscopic evaluation to assess for vocal fold dysfunction. Second, laterality is critical to the assessment—bilateral vocal fold paralysis is more likely to necessitate definitive airway intervention, whereas unilateral paralysis may allow for compensatory mechanisms but still carry substantial aspiration risk. However, regardless of laterality, vocal fold paralysis in Williams syndrome can significantly impair swallowing and may necessitate gastrostomy tube placement. In light of this, multidisciplinary care becomes essential: optimal outcomes require coordinated involvement of otolaryngology, gastroenterology, genetics, speech‐language pathology, and others.

These additional patient cases add to the body of knowledge regarding vocal fold complications in Williams syndrome patients so that providers can be aware of such findings that have important long‐term implications. Additionally, they highlight the importance of considering genotype‐specific mechanisms when evaluating atypical airway and feeding manifestations in Williams syndrome and underscore how laterality of vocal fold paralysis directly influences management strategies and long‐term outcomes. Further study is warranted in order to characterize the disturbances in vocal cord function and phonation that can be observed within Williams syndrome patients as a result of elastin haploinsufficiency, as well as other genetic contributors. Future steps may involve targeted gene sequencing that can more precisely identify which gene deletions can result in vocal fold paralysis.

This report is limited by its small sample size and retrospective nature, which restricts generalizability. Additionally, while genetic findings were identified in both patients, causal relationships between specific gene deletions and vocal fold paralysis remain speculative. Variability in clinical management and follow‐up duration between cases further limits direct comparison of outcomes. Larger studies are needed to better characterize the prevalence, mechanisms, and long‐term prognosis of vocal fold dysfunction in Williams syndrome.

## 5. Conclusion

Williams syndrome patients are complex, requiring multidisciplinary care for their management. The patients detailed in this case report exhibited many of the expected manifestations of the syndrome, while additionally presenting with an atypical finding of unilateral or bilateral vocal cord palsies. We anticipate this dysfunction as a result of the region of chromosomal deletion involving the elastin gene and present these cases to inform providers of this potential finding when caring for these patients.

## Author Contributions

Priya Arya: original draft writing and review and editing.

Miller C. Hamrick: review and editing.

Benjamin Mackowiak: review and editing.

Bradley Buckler: review and editing.

Chad Haldeman‐Englert: review and editing.

Stephanie E. Ambrose: conceptualization, original draft writing, and review and editing.

## Funding

The authors have nothing to report.

## Disclosure

The authors declare that there are no additional disclosures to report.

## Ethics Statement

This study was conducted in accordance with institutional ethical standards. The study was deemed exempt from IRB approval due to its retrospective case report design involving minimal risk and no direct patient interaction. Informed consent for publication of clinical details was obtained from the patients’ legal guardians. All efforts were made to ensure patient anonymity and confidentiality.

## Conflicts of Interest

The authors declare no conflicts of interest.

## Data Availability

Data sharing is not applicable to this article as no datasets were generated or analyzed during the current study.
